# Reductions in AFP and PIVKA-II can predict the efficiency of anti-PD-1 immunotherapy in HCC patients

**DOI:** 10.1186/s12885-021-08428-w

**Published:** 2021-07-04

**Authors:** Xuqi Sun, Jie Mei, Wenping Lin, Ziliang Yang, Wei Peng, Jinbin Chen, Yaojun Zhang, Li Xu, Minshan Chen

**Affiliations:** 1 Sun Yat-Sen University Cancer Center, State Key Laboratory of Oncology in South China; Collaborative Innovation Center for Cancer Medicine, Guangzhou, 510060 China; 2 Department of Liver Surgery, Sun Yat-Sen University Cancer Center, Dongfeng East Road 651, Guangzhou, 510060 China; 3 Zhongshan School of Medicine, Sun Yat-Sen University, Guangzhou, 510060 China

**Keywords:** Hepatocellular carcinoma, AFP, PIVKA-II, Immunotherapy, Survival

## Abstract

**Background:**

Few biomarkers can predict the efficiency of PD-1 blockade in patients with hepatocellular carcinoma (HCC). This study aimed to investigate the prognostic role of AFP and PIVKA-II in HCC patients receiving anti-PD-1 immunotherapy.

**Methods:**

A total of 235 HCC patients treated with PD-1 blockade were enrolled. Serum AFP and PIVKA-II levels were collected before and after treatments. The patients were divided into groups based on the reduction in AFP and PIVKA-II: AFP reduction ≤50% vs AFP reduction > 50% and PIVKA-II reduction ≤50% vs PIVKA-II reduction > 50%. The primary endpoints included objective response rate (ORR), progression-free survival (PFS) and overall survival (OS). Binary logistic regression analyses were used to explore the related factors of ORR. A Cox proportional hazards model was employed to identify the potential prognostic factors of PFS and OS.

**Results:**

Among all the patients, 34.9% (82/235) achieved a complete or partial response. There was a positive correlation between AFP reduction > 50% or PIVKA-II reduction> 50% and the ORR of PD-1 blockade (*P* < 0.001 and = 0.003). PFS was significantly improved in patients with AFP reduction > 50% and PIVKA-II reduction > 50% (*p* < 0.001 and = 0.021). In addition, AFP reduction > 50% and PIVKA-II reduction> 50% were positively correlated with longer OS (*p* = 0.003 and 0.006).

**Conclusion:**

Early reductions in AFP and PIVKA-II can be predictors of the efficacy of PD-1 blockade in HCC patients.

## Introduction

Hepatocellular carcinoma (HCC) ranks as the sixth most common malignancies and fourth leading cause of cancer-related mortality worldwide [1, 2]. Due to the insidious onset of HCC, approximately 80% of HCC patients are diagnosed at an advanced stage [3, 4]. Although sorafenib and lenvatinib are approved as the first-line treatment for advanced HCC, the survival of these patients remains dismal [5]. In recent years, the emergence of anti-programmed death 1 (PD-1) checkpoint inhibitors has changed the landscape of systemic treatments for advanced HCC. The objective response rates (ORRs) can reach to 17–20% in advanced HCC patients who receive anti-PD-1 therapy as monotherapy [6, 7]. Furthermore, the combination of atezolizumab and bevacizumab (A + T) achieved significantly longer survival in HCC patients than sorafenib, which increased the first-line treatments for advanced HCC [8]. Although current studies indicate promising efficiency of anti-PD-1 therapy, the ORR remains unsatisfactory. Even with the A + T protocol, the ORR is only 27.3% [8]. How to identify potential patients who would respond to anti-PD-1 therapy remains to be solved.

Unfortunately, HCC lacks efficient biomarkers to predict the efficiency of anti-PD-1 therapy. Alpha-fetoprotein (AFP) and protein induced by vitamin K absence or antagonist-II (PIVKA-II) are common diagnostic and prognostic biomarkers for HCC that have a positive correlation with tumor burden [9]. AFP and PIVKA-II can also predict the recurrence and survival of HCC patients [10]. For patients treated with sorafenib, an early decrease in serum AFP levels indicates a higher probability of response [11]. In HCC patients receiving transarterial chemoembolization (TACE), the response of AFP and PIVKA-II is positively associated with the radiological response [12, 13]. However, the predictive role of AFP and PIVKA-II in HCC patients receiving anti-PD-1 therapy remains unclear.

In this study, we aimed to assess the predictive role of early reduction in AFP and PIVKA-II for HCC patients receiving anti-PD-1 therapy. The primary endpoints included ORR, progression-free survival (PFS) and overall survival (OS). These results can assist in identifying potential HCC patients responding to anti-PD-1 therapy, which improves the effective utilization rates of anti-PD-1 immunotherapy.

## Methods

### Patients

We retrospectively reviewed HCC patients receiving anti-PD-1 therapy at Sun Yat-sen University Cancer Center from January 1, 2018 to December 31, 2019. The inclusion criteria were as follows: 1) clinically or pathologically diagnosed with HCC according to NCCN guidelines; 2) age at diagnosis ≥18 years; and 3) treatment with at least one dose of anti-PD-1 therapy. We initially enrolled 619 patients into study. The exclusion criteria were as follows: 1) no baseline imaging records before anti-PD-1 therapy (*n* = 203); 2) no baseline AFP or PIVKA-II levels before anti-PD-1 therapy (*n* = 35); 3) no follow-up imaging or tumor marker records after anti-PD-1 therapy (*n* = 66); 4) no elevated baseline AFP or PIVKA-II levels (AFP ≤ 25 ng/ ml or PIVKA-II ≤ 40 mAU/ml) (*n* = 80) and 5) taking anticoagulants (*n* = 0). Eventually, 235 HCC patients were included for analysis. None of these patients were from early phase clinical trials. PD-1 blockades were intravenously administered at the standard dose as follows: pembrolizumab 200 mg, nivolumab 100 mg, toripalimab 240 mg, camrelizumab 200 mg or sintilimab 200 mg every 3 weeks. The median number of courses of PD-1 blockades was four (range, 1–19). The adverse events (AEs) were evaluated based on the Common Terminology Criteria for Adverse Events v5.0. Patients were treated according to the treatments plan until intolerable AEs occurred or the disease progressed. This study was approved by the Ethics Committee of Sun Yat-sen University Cancer Center.

### Patient follow-up

The serum levels of AFP and PIVKA-II were measured within 7 days before the first dose of anti-PD-1 therapy. To evaluate the change in AFP and PIVKA-II levels after immunotherapy, we further collected AFP and PIVKA-II data after 6 ± 1 weeks of anti-PD-1 therapy. During treatment, patients underwent abdominal contrast enhanced computer tomography (CT) or magnetic resonance imaging and chest enhanced CT every 6–8 weeks. The tumor response was evaluated according to Response Evaluation Criteria in Solid Tumors (RECIST 1.1) [14]. Complete response (CR) was defined as the disappearance of all the targeted lesions. Partial response (PR) was defined as at least a 30% reduction in the sum of diameters of targeted lesions. The association between AFP/PIVKA-II reduction and ORR/PFS/OS was assessed. Based on previous studies, a reduction in AFP/PIVKA-II serum concentration > 50% was adopted as the cutoff value for serum response. The definitions of primary endpoints in this study were as follows: 1) ORR, the proportion of HCC patients achieving CR or PR; 2) PFS, the period from the date of first dose of anti-PD-1 therapy to the date of progressive disease (PD), death or last follow-up; and 3) OS, the time during the date of first dose of anti-PD-1 therapy to the date of death or last follow-up.

### Statistical analysis

Categorical variables are expressed as numerical values with percentages. The chi-square test was employed to evaluate the correlation between the reduction in AFP/PIVKA-II and ORR. Binary logistic regression analysis was performed to identify potential predictors for ORR, including age at diagnosis, gender, vascular invasion, extrahepatic metastasis, albumin-bilirubin (ALBI) grade [15], AFP reduction, PIVKA-II reduction, baseline AFP level and baseline PIVKA-II level. Odds ratios (ORs) were calculated for each variable in the logistical model. The PFS and OS were compared by the Kaplan-Meier method with the log-rank test. Hazard ratios (HRs) for OS and PFS were calculated by the Cox regression model. A multivariate Cox regression model was adopted to evaluate the significance of clinical factors that were statistically significant in the univariate analyses. Receiver operating characteristic (ROC) curves were plotted to compare the performance of AFP reduction, PIVKA-II reduction and AFP-ALBI-PIVKA-II score for predicting ORR, and the DeLong Method was performed to compare the area under the ROC curves (AUROC). Moreover, we also calculated the Akaike information criterion (AIC) of all the prognostic models. A two-tailed *P* value less than 0.05 was statistically significant. All analyses were performed with the IBM SPSS, version 26.0 and R software version 3.6.1.

## Results

### Patient characteristics

The baseline characteristics of 235 HCC patients are listed in Table 1. The median age of this cohort was 51.0 years (range, 21–84 years). The majority of patients were infected with hepatitis B virus (86.4%). For patients with HBV infection, 72.9% (148/203) received Entecavir and 27.1% (55/203) received Tenofovir. Among these patients, 51.9% (122/235) had vascular invasion, and 37.0% (87/235) had extrahepatic metastasis. Most patients had good liver function, and 97.0% (228/235) were Child-Pugh A class while the other seven patients were Child-Pugh B class. The median baseline level was 2995.0 ng/ml for AFP and 7209.0 mAU/ml for PIVKA-II. After 6 weeks of anti-PD-1 immunotherapy, the AFP level of 48.1% (113/235) of patients decreased by more than 50% from baseline, and the PIVKA-II level of 53.2% (125/235) of patients decreased by more than 50% from baseline. Among all the patients, 7.7% (18/235) received anti-PD-1 therapy as monotherapy, 22.1% (52/235) received anti-PD-1 therapy plus targeted drugs, 30.2% (71/235) received anti-PD-1 therapy plus locoregional treatments, including TACE or hepatic arterial infusion chemotherapy, and 40.0% (94/235) received anti-PD-1 therapy combined with targeted drugs and locoregional treatments. The majority of patients received anti-PD-1 immunotherapy as the first-line treatment (91.9%), and 8.1% (19/235) were treated with PD-1 blockades as the second-line therapy. During follow-up, all the AEs were manageable, and no toxicity-related death occurred.

**Table 1:** Baseline characteristics of HCC patients receiving anti-PD-1 blockades

Characteristics	Number (%)
Sample size	235
Age, years	
≤ 50	109 (46.4)
>50	126 (53.6)
Chronic liver disease	
HBV	203 (86.4)
None	32 (13.6)
Gender	
Female	31 (13.2)
Male	204 (86.8)
Vascular invasion	
No	113 (48.1)
Yes	122 (51.9)
Extrahepatic metastasis	
No	148 (63.0)
Yes	87 (37.0)
ALBI grade	
I	142 (60.4)
II	93 (39.6)
AFP reduction> 50%	
No	122 (51.9)
Yes	113 (48.1)
PIVKA-II reduction> 50%	
No	110 (46.8)
Yes	125 (53.2)
Baseline AFP level, ng/ml	
Median (range)	2995.0 (25.14–121,000)
≤ 400	78 (33.2)
> 400	157 (66.8)
Baseline PIVKA-II level, mAU/ml	
Median (range)	7209.0 (41–75,000)
≤ 400	44 (18.7)
> 400	191 (81.3)

### Correlation between AFP or PIVKA-II levels and ORR

Of 235 enrolled HCC patients, 3.4% (8/235) patients achieved CR and 31.5% (74/235) achieved PR after anti-PD-1-based treatments. The chi-square tests revealed a significant correlation between the reduction in AFP or PIVKA-II and the ORR of HCC patients. For patients with AFP reduction> 50, 53.1% (60/113) responded to anti-PD-1 therapy, while only 18.0% (22/122) patients responded to immunotherapy in those with AFP reduction ≤50% (*p* < 0.001). In terms of PIVKA-II, 49.6% (62/125) of patients achieved a response to anti-PD-1-based treatments in those with PIVKA-II reduction > 50%, while only 18.2% (20/110) responded to anti-PD-1 therapy in patients with PIVKA-II reduction ≤50% (*p* < 0.001). After binary logistic analyses, there was no significant association between ORR and age at diagnosis or gender (*p* = 0.405 and 0.128). Vascular invasion had no significant impact on the ORR (*p* = 0.138). Although extrahepatic metastasis showed an adverse impact on the ORR in univariate analysis, no significant correlation was found in the multivariate analysis (*p* = 0.112). For the baseline level of serum markers, neither AFP nor PIVKA-II had a significant correlation with the ORR (*p* = 0.131 and 0.354). The ALBI grades were negatively associated with ORR (*p* = 0.025). After multivariate analyses, AFP reduction > 50% was positively related to higher ORRs (*p* < 0.001). Similarly, PIVKA-II reduction > 50% was also an independent factor for ORR (*p* = 0.003). Detailed data are listed in Table 2.

**Table 2:** Univariate and multivariate analyses for objective response rates (ORR)

Variables	Univariate		Multivariate	
	OR (95%CI)	*P* value	OR (95%CI)	*P* value
Age, years		0.405		
≤ 50	1.0			
>50	1.26 (0.73–2.16)			
Gender		0.128		
Female	1.0			
Male	2.0 (0.82–4.85)			
Vascular invasion		0.138		
No	1.0			
Yes	1.51 (0.88–2.59)			
Extrahepatic metastasis		0.002		0.112
No	1.0		1.0	
Yes	0.38 (0.21–0.69)		0.58 (0.30–1.14)	
ALBI grade		0.038		0.025
I	1.0		1.0	
II	0.55 (0.31–0.97)		0.49 (0.26–0.91)	
AFP reduction> 50%		<0.001		< 0.001
No	1.0		1.0	
Yes	5.15 (2.85–9.29)		3.36 (1.75–6.45)	
PIVKA-II reduction> 50%		<0.001		0.003
No	1.0		1.0	
Yes	4.43 (2.44–8.05)		2.72 (1.41–5.26)	
Baseline AFP level > 400 ng/ml		0.131		
No	1.0			
Yes	1.58 (0.87–2.84)			
Baseline PIVKA-II level > 400 mAU/ml		0.354		
No	1.0			
Yes	0.73 (0.37–1.43)			

### The prognostic role of AFP and PIVKA-II in PFS

**Fig. 1:**
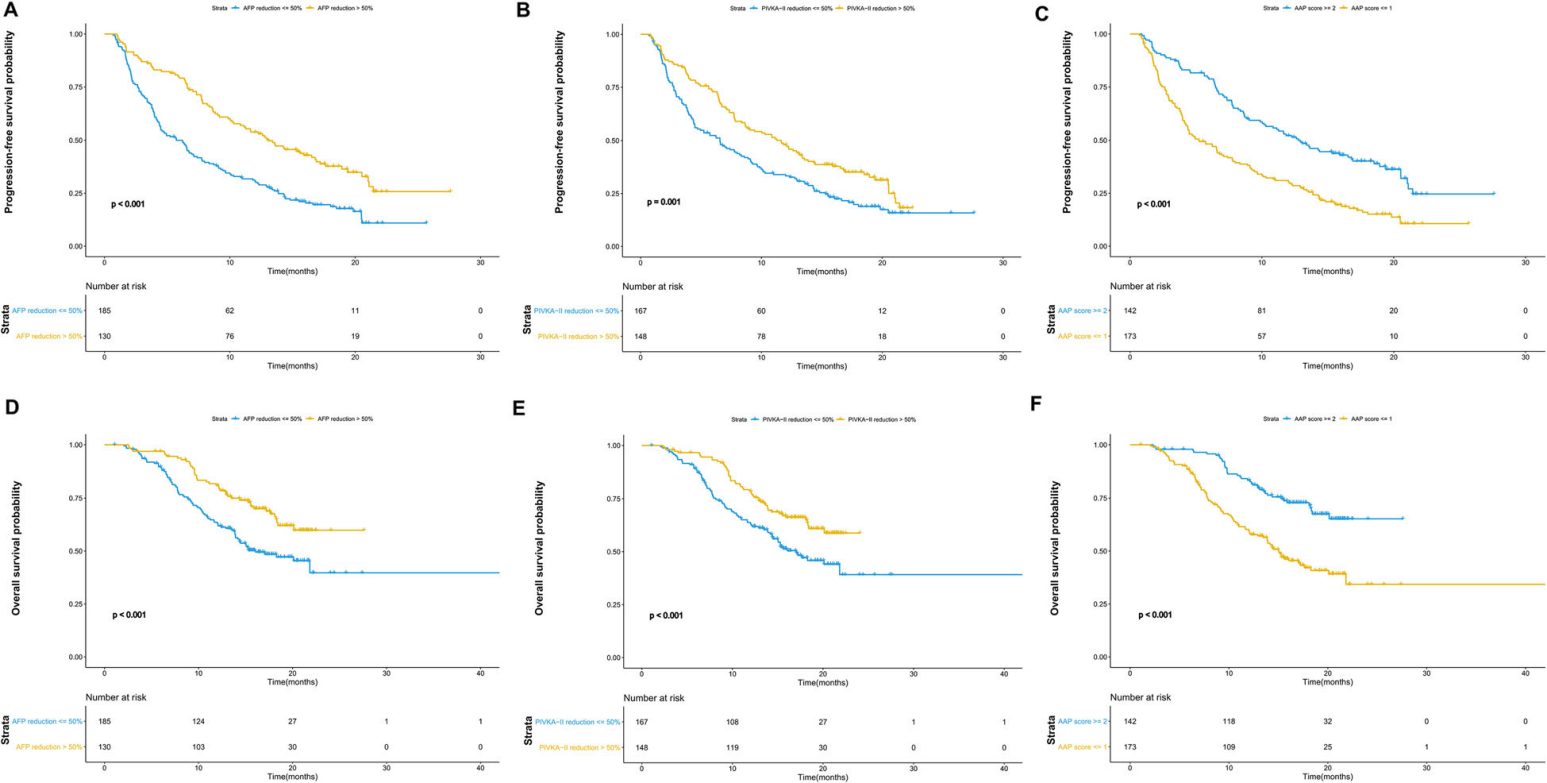
The survival curves of HCC patients receiving anti-PD-1 immunotherapy. Patients with AFP reduction> 50% has significantly longer progression-free survival (PFS) than those with AFP reduction≤50% (A). Similarly, patients with PIVKA-II reduction > 50% also had better PFS (B). The PFS of HCC patients with AAP score ≥ 2 was significantly improved than those with AAP score ≤ 1 (C). In terms of overall survival (OS), patients with AFP reduction> 50% has significantly improved OS compared to those without (D). The OS of patients with PIVKA-II reduction > 50% was significantly longer (E). Patients with AAP score ≤ 1 had worse OS compared to those with AAP score ≥ 2 (F)

The median PFS was 7.7 months (95% confidence interval (CI) 6.3–9.2 months). Age, gender and baseline level of PIVKA-II had no significant correlation with PFS. The survival curves of PFS are shown in Fig. 1A and B. The PFS of HCC patients with AFP reduction > 50% was significantly longer than that of patients with AFP reduction ≤50% (13.1 months vs 4.5 months, *p* < 0.001). Similarly, HCC patients with PIVKA-II reduction > 50% had longer PFS than those with PIVKA-II reduction≤50% (10.9 months vs 4.5 months, *p* = 0.001). After multivariate analyses, vascular invasion and extrahepatic metastasis had a significant adverse impact on PFS (*p* < 0.001 and = 0.014). A higher ALBI grade was associated with worse PFS (p < 0.001). Patients with AFP reduction > 50% or PIVKA-II reduction > 50% had improved PFS compared to those with serum marker reduction ≤50% (*p* < 0.001 and = 0.021). A higher baseline level of AFP was associated with worse PFS (*p* = 0.001) (Table 3).

**Table 3:** Univariate and multivariate analyses for progression-free survival (PFS)

Variable	Univariate		Multivariate	
	HR (95%CI)	*P* value	HR (95%CI)	*P* value
Age, years		0.138		0.008
≤ 50	1.0		1.0	
>50	0.75 (0.51–1.10)		0.69 (0.52–0.91)	
Gender		0.780		
Female	1.0			
Male	1.09 (0.59–2.04)			
Vascular invasion		0.025		< 0.001
No	1.0		1.0	
Yes	1.57 (1.06–2.33)		2.19 (1.42–3.36)	
Extrahepatic metastasis		0.001		0.014
No	1.0		1.0	
Yes	1.97 (1.34–2.91)		1.67 (1.11–2.49)	
ALBI grade		< 0.001		<0.001
I	1.0		1.0	
II	2.13 (1.45–3.13)		2.04 (1.39–3.00)	
AFP reduction> 50%		<0.001		<0.001
No	1.0		1.0	
Yes	0.37 (0.25–0.56)		0.38 (0.23–0.61)	
PIVKA-II reduction> 50%		0.001		0.021
No	1.0		1.0	
Yes	0.46 (0.31–0.68)		0.60 (0.39–0.93)	
Baseline AFP level > 400 ng/ml		< 0.001		0.001
No	1.0		1.0	
Yes	2.37 (1.49–3.77)		2.25 (1.41–3.59)	
Baseline PIVKA-II level > 400 mAU/ml		0.164		
No	1.0			
Yes	1.47 (0.85–2.55)			

HR, hazard ratio for progression-free survival.

### The prognostic role of AFP and PIVKA-II in OS

The median OS was 20.1 months (95% CI, 17.1–23.1 months). Patients with AFP reduction > 50% had significantly longer OS than those without (not reached vs 13.7 months, *p* < 0.001) (Fig. 1D). In terms of PIVKA-II, a reduction> 50% had a positive impact on OS (not reached vs 14.4 months, *p* < 0.001) (Fig. 1E). Similar to PFS, there was no significant association between OS and age, gender or the baseline level of PIVKA-II. The baseline level of AFP was negatively correlated with OS (*p* = 0.001). Patients with vascular invasion or extrahepatic metastasis had worse OS than those without (p = 0.001 and 0.038). Additionally, a higher ALBI grade adversely affected the OS (*P* < 0.001). The OS of patients with AFP reduction > 50% or PIVKA-II reduction > 50% was significantly longer than that of patients without AFP or PIVKA-II reduction (*p* = 0.003 and 0.006) (Table 4).

**Table 4:** Univariate and multivariate analyses for overall survival (OS)

Variable	Univariate		Multivariate	
	HR (95%CI)	*P* value	HR (95%CI)	*P* value
Age, years		0.202		
≤ 50	1.0			
>50	0.78 (0.53–1.14)			
Gender		0.411		
Female	1.0			
Male	1.30 (0.70–2.43)			
Vascular invasion		0.007		0.001
No	1.0		1.0	
Yes	1.73 (1.17–2.57)		2.09 (1.38–3.18)	
Extrahepatic metastasis		0.006		0.038
No	1.0		1.0	
Yes	1.72 (1.17–2.54)		1.53 (1.02–2.30)	
ALBI grade		< 0.001		<0.001
I	1.0		1.0	
II	2.35 (1.59–3.45)		2.28 (1.54–2.37)	
AFP reduction> 50%		< 0.001		0.003
No	1.0		1.0	
Yes	0.44 (0.30–0.66)		0.50 (0.32–0.80)	
PIVKA-II reduction> 50%		< 0.001		0.006
No	1.0		1.0	
Yes	0.49 (0.33–0.72)		0.54 (0.35–0.84)	
Baseline AFP level > 400 ng/ml		0.001		0.001
No	1.0		1.0	
Yes	2.27 (1.43–3.62)		2.31 (1.44–3.70)	
Baseline PIVKA-II level > 400 mAU/ml		0.142		
No	1.0			
Yes	1.51 (0.87–2.61)			

HR, hazard ratio for overall survival.

### The prognostic value of the AAP score

**Fig. 2:**
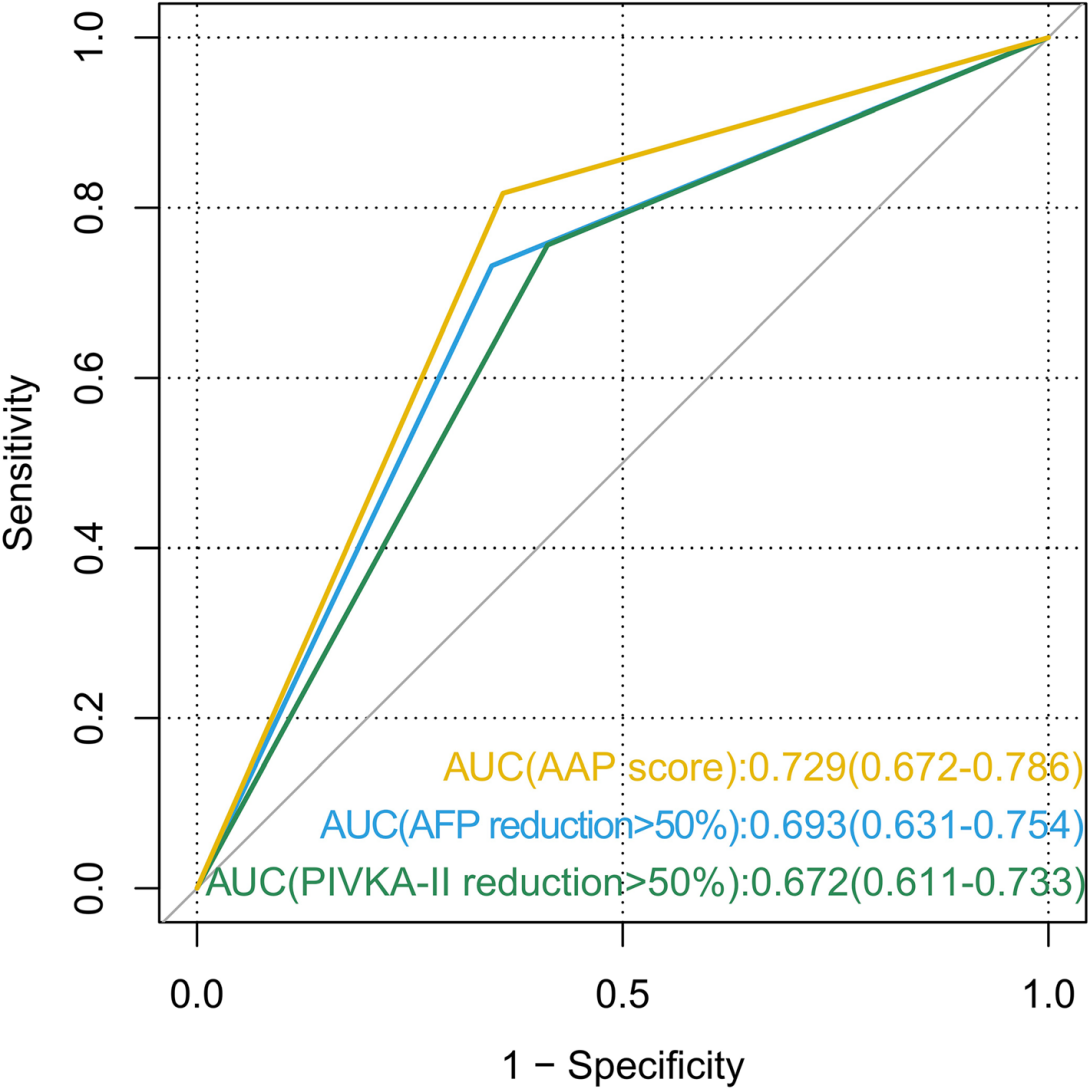
Receiver operating characteristics curves of AFP reduction, PIVKA-II reduction and the AAP score for identifying response (CR + PR) of HCC patients treated with anti-PD-1 immunotherapy

We developed an AFP-ALBI-PIVKA-II (AAP) score according to the independent predictors for ORR. The AAP score consisted of three variables: AFP reduction, ALBI grade and PIVKA-II reduction. The AAP score was calculated as follows: AFP reduction> 50% (yes = 1, no = 0), ALBI grade (I = 1, II = 0), and PIVKA-II reduction> 50% (yes = 1, no = 0). Patients were further stratified into two groups based on their AAP score. A total of 51.9% (122/235) of patients had AAP scores ≥2 and 48.1% (113/235) of patients had AAP scores ≤1. The PFS and OS were significantly longer in patients with AAP scores ≥2 than in those with scores ≤1 (both *p* < 0.001) (Fig. 1C and F). We also compared the AUROCs of different variables for predicting the response to anti-PD-1 therapy (Fig. 2). The AUROC for AFP reduction was 0.693 (95% CI, 0.631–0.754). The AUROC for PIVKA-II reduction was 0.672 (95% CI, 0.611–0.733). The AAP score had the best predictive performance, with an AUROC of 0.729 (95% CI, 0.672–0.786). In the pairwise comparison of AUROCs, there was no significant difference between AFP reduction and PIVKA-II reduction (*p* = 0.596). Although the AUROCs was not statistically different between AFP reduction and AAP score (*p* = 0.179), the accuracy of AAP score was significantly better than PIVKA-II reduction (*p* = 0.038). The results of AIC indicated that the AAP score had superior prognostic ability than AFP reduction and PIVKA-II reduction, with the AIC values being 260.44, 275.36 and 281.59 respectively.

## Discussion

To date, no effective biomarkers have been identified to predict the efficiency of anti-PD-1 therapy in HCC patients. In this study, we found that after 6 weeks of anti-PD-1 immunotherapy, an AFP or PIVKA-II reduction > 50% from the baseline was significantly associated with a better response and improved survival. These results can assist in identifying HCC patients who may not benefit from anti-PD-1 therapy and making timely adjustments to treatment regimens.

In recent years, immunotherapy, including anti-PD-1 therapy, has changed the landscape of systemic treatments for advanced HCC. Encouraged by the promising efficiency of nivolumab and pembrolizumab as monotherapies in HCC, oncologists are making efforts to explore how to improve the response to anti-PD-1 therapy by combining immunotherapy with other treatment modalities [16–18]. However, even atezolizumab (anti-PD-L1 antibody) plus bevacizumab (anti-vascular endothelial growth factor antibody) achieved better clinical outcomes than sorafenib, and the ORR of this combination regimen was only 27.3% [8]. Furthermore, most HCC patients suffer from primary or secondary resistance to anti-PD-1 immunotherapy during treatments [19]. In this context, how to identify potential HCC patients responding to anti-PD-1 therapy needs to be solved urgently. Unfortunately, there is a lack of predictive biomarkers for the response of HCC patients to anti-PD-1 immunotherapy. Studies have reported the association between peripheral blood markers and the efficiency of anti-PD-1 therapy. In melanoma, the baseline level and change in white blood cells (WBCs), lactate dehydrogenase (LDH) and C-reactive protein (CRP) can predict the response and survival of patients treated with anti-PD-1 immunotherapy [20, 21]. For patients with advanced non-small cell lung cancer (NSCLC), high levels of WBCs and eosinophils have a positive impact on the survival of patients receiving nivolumab [22]. Until now, predictive biomarkers for the efficiency of immunotherapy have been limited in HCC. Studies have shown that the neutrophil lymphocyte ratio (NLR) and tumor growth factor-β (TGF-β) affect the survival of HCC patients receiving anti-PD-1 immunotherapy [23, 24]. Although AFP and PIVKA-II are extensively used for HCC, few studies have assessed their predictive value in HCC patients treated with anti-PD-1 immunotherapy.

AFP has been widely used for surveillance and noninvasive diagnosis of HCC for several decades. Its predictive role in the prognosis of HCC patients has also been validated [9]. PIVKA-II is abnormal prothrombin, which is induced by carboxylation dysfunction of N-terminal glutamic acid residues [25]. Numerous studies have confirmed its clinical utility in HCC. High serum levels of PIVKA-II are associated with more aggressive tumor behavior [25]. The baseline and change in PIVKA-II during treatments can predict the prognosis of HCC patients [25]. Although there is no significant correlation between the serum levels of AFP and PIVKA-II, both of them can reflect the tumor burden of HCC patients [9]. The predictive roles of AFP and PIVKA-II have been validated in HCC patients treated with locoregional therapy or targeted drugs. Researchers have found that the reduction in AFP and PIVKA-II can help to assess the response of patients to HAIC [26]. For advanced HCC patients treated with TACE, patients with AFP and PIVKA-II reduction > 50% after 3 months of TACE had a better prognosis than those without [27]. Kodama et al. found that early decreases in AFP and PIVKA-II are positively associated with the imaging response to lenvatinib [28]. Based on these studies, it can be inferred that the serum response to AFP and PIVKA-II can reflect the response of HCC patients to anti-PD-1 immunotherapy. In patients with NSCLC, the reduction in common lung cancer markers, including carcinoembryonic antigen and cytokeratin fragment 19, are both reliable predictive markers for immunotherapy in NSCLC patients [29]. In accordance with NSCLC, the results showed that the reduction in HCC markers, including AFP and PIVKA-II, can also predict the efficiency of anti-PD-1 therapy and the prognosis of HCC patients.

In this study, we also found patients with higher ALBI grade had lower response rates. Cirrhosis is the common concomitant liver disease in HCC patients, and higher ALBI grade indicates worse liver function and more severe cirrhosis [15]. Cirrhosis can assist tumor immune escape and induce immunosuppressive microenvironment. For example, cirrhotic livers have higher expression of extracellular matrix, which can further suppress the anti-tumor immunity by activating the transforming growth factor beta [30, 31]. Moreover, liver fibrogenesis is induced by hepatic stellate cells, which can also decrease the infiltration of lymphocytes and increase the proliferation of immunosuppressive cells [32]. Jeffrey et al. have also found HCC patients with patients with lower ALBI grades had better response to immunotherapy and prognosis [33]. Our results were similar with the above findings and indicated that the response to immunotherapy might be affected by the severity of cirrhosis.

Although it is generally recognized that imaging methods can evaluate the efficiency of treatments for HCC patients, there exist limitations for imaging. For instance, in patients receiving radiofrequency ablation, TACE or radiotherapy, a considerable number of patients can achieve tumor necrosis, decreased tumor activity, or even pathological remission, but imaging methods may not reflect these changes in tumor burden. In addition, the inflammation or edema caused by antitumor treatment can obstruct imaging methods to reflect changes in tumors [34]. These deficiencies indicate that other evaluation methods should be applied for assisting imaging methods to assess the anti-tumor efficiency more accurately. AFP and PIVKA-II are commonly recognized predictors for the survival of HCC patients receiving non-radical treatments [9]. In this study, we found that after 6 weeks of anti-PD-1 therapy, an AFP or PIVKA-II early reduction > 50% from baseline can predict better survival of HCC patients, and the serum response also showed good correlation with imaging response. According to the ORR results, we further developed an AAP score consisting of ALBI grade, AFP reduction and PIVKA-II reduction to predict the efficiency of anti-PD-1 immunotherapy. The AUC values showed that the AAP score had better predictive performance than a single factor.

There are several limitations in this study except for its retrospective nature. First, this is a single-center study enrolling HCC patients receiving anti-PD-1 immunotherapy. Due to the requirements of indications for PD-1 antibody, not all HCC patients were enrolled. There might exist potential biases in the selection of patients. This study focused on patients with advanced HCC who are the targeted population of anti-PD-1 therapy. In addition, a single-center study could be an advantageous factor to ensure the consistency of the assessment of clinical and survival data before and during treatment. Second, the median OS of HCC patients with AFP or PIVKA-II reduction > 50% was not reached, so further follow-up is expected. However, this study validated the significant association between the early reduction in HCC markers and PFS, and these results can assist oncologists in making better treatment decisions on anti-PD-1 immunotherapy. For instance, in HCC patients with poor survival, if no imaging response is observed after anti-PD-1 immunotherapy and the serum levels of AFP and PIVKA-II do not decrease, there might be no need to continue to use anti-PD-1 immunotherapy. Third, this study enrolled relatively mixed patients because patients received various kinds of anti-PD-1-based treatments. However, this cohort could better represent the real-world HCC population. In recent years, anti-PD-1-based treatments have improved the prognosis of HCC patients. For instance, the combination of PD-1 antibody and targeted drugs can significantly improve the survival of HCC patients, and the A + T regimen has been one of the first-line treatments for advanced HCC patients [8]. Jie et al. has found that PD-1 antibody plus locoregional therapy can improve the efficiency of anti-PD-1 immunotherapy [35]. Besides, anti-PD-1 therapy combined with targeted drugs and locoregional therapy can further prolong the survival of HCC patients compared with lenvatinib monotherapy [36]. Till now, no randomized controlled trial has been performed to compare the efficiency of these treatments, so the clinical treatments are mainly based on oncologists and patients’ preference. Multicenter prospective randomized studies are expected to further validate these findings.

In conclusion, this study indicated that the early reduction in AFP and PIVKA-II had a positive association with the response of HCC patients to anti-PD-1 immunotherapy. After 6 weeks of anti-PD-1 therapy, an AFP or PIVKA-II reduction > 50% indicated prolonged PFS and OS. Monitoring the serum levels of AFP and PIVKA-II could help to assess and predict the efficiency of immunotherapy for HCC patients.

## Data Availability

The datasets used during the current study are available from the corresponding author on reasonable request.

## Abbreviations

HCC: Hepatocellular carcinoma
PD-1: Anti-programmed death 1
ORR: Objective response rate
A + T: Atezolizumab and bevacizumab
AFP: Alpha-fetoprotein
PIVKA-II: Protein induced by vitamin K absence or antagonist-II
TACE: Transarterial chemoembolization
PFS: Progression-free survival
OS: Overall survival
AE: Adverse event
CT: Computer tomography
RECIST: Response Evaluation Criteria in Solid Tumors
CR: Complete response
PR: Partial response
PD: Progressive disease
ALBI: Albumin-bilirubin
OR: Odds ratio
ROC: Receiver operating characteristic
HAIC: Hepatic arterial infusion chemotherapy
CI: Confidence interval
AAP: AFP-ALBI-PIVKA-II
AUC: Area under the curve
WBC: White blood cell
LDH: Lactate dehydrogenase
CRP: C-reactive protein
NSCLC: Non-small cell lung cancer
NLR: Neutrophil lymphocyte ratio
TGF-β: Tumor growth factor-β

## References

[1] Bray F, Ferlay J, Soerjomataram I, Siegel RL, Torre LA, Jemal A (2018). Global cancer statistics 2018: GLOBOCAN estimates of incidence and mortality worldwide for 36 cancers in 185 countries. CA Cancer J Clin.

[2] Siegel RL, Miller KD, Jemal A (2020). Cancer statistics, 2020. CA Cancer J Clin.

[3] Forner A, Reig M, Bruix J (2018). Hepatocellular carcinoma. Lancet.

[4] Li D, Sedano S, Allen R, Gong J, Cho M, Sharma S. Current Treatment Landscape for Advanced Hepatocellular Carcinoma: Patient Outcomes and the Impact on Quality of Life. Cancers (Basel). 2019;11(6). 10.3390/cancers11060841. 10.3390/cancers11060841 PMC6627588 31216701

[5] Kudo M, Finn RS, Qin S, Han KH, Ikeda K, Piscaglia F, Baron A, Park JW, Han G, Jassem J, Blanc JF, Vogel A, Komov D, Evans TRJ, Lopez C, Dutcus C, Guo M, Saito K, Kraljevic S, Tamai T, Ren M, Cheng AL (2018). Lenvatinib versus sorafenib in first-line treatment of patients with unresectable hepatocellular carcinoma: a randomised phase 3 non-inferiority trial. Lancet.

[6] El-Khoueiry AB, Sangro B, Yau T, Crocenzi TS, Kudo M, Hsu C, Kim TY, Choo SP, Trojan J, Welling THR (2017). Nivolumab in patients with advanced hepatocellular carcinoma (CheckMate 040): an open-label, non-comparative, phase 1/2 dose escalation and expansion trial. Lancet.

[7] Zhu AX, Finn RS, Edeline J, Cattan S, Ogasawara S, Palmer D, Verslype C, Zagonel V, Fartoux L, Vogel A, Sarker D, Verset G, Chan SL, Knox J, Daniele B, Webber AL, Ebbinghaus SW, Ma J, Siegel AB, Cheng AL, Kudo M, Alistar A, Asselah J, Blanc JF, Borbath I, Cannon T, Chung K, Cohn A, Cosgrove DP, Damjanov N, Gupta M, Karino Y, Karwal M, Kaubisch A, Kelley R, van Laethem JL, Larson T, Lee J, Li D, Manhas A, Manji GA, Numata K, Parsons B, Paulson AS, Pinto C, Ramirez R, Ratnam S, Rizell M, Rosmorduc O, Sada Y, Sasaki Y, Stal PI, Strasser S, Trojan J, Vaccaro G, van Vlierberghe H, Weiss A, Weiss KH, Yamashita T (2018). Pembrolizumab in patients with advanced hepatocellular carcinoma previously treated with sorafenib (KEYNOTE-224): a non-randomised, open-label phase 2 trial. Lancet Oncol.

[8] Finn RS, Qin S, Ikeda M, Galle PR, Ducreux M, Kim TY, Kudo M, Breder V, Merle P, Kaseb AO, Li D, Verret W, Xu DZ, Hernandez S, Liu J, Huang C, Mulla S, Wang Y, Lim HY, Zhu AX, Cheng AL (2020). Atezolizumab plus Bevacizumab in Unresectable Hepatocellular Carcinoma. N Engl J Med.

[9] Park H, Park JY (2013). Clinical significance of AFP and PIVKA-II responses for monitoring treatment outcomes and predicting prognosis in patients with hepatocellular carcinoma. Biomed Res Int.

[10] Iwadou S, Nouso K, Kuwaki K, Kobayashi Y, Nakamura S, Tanaka H, Miyoshi K, Ohnishi H, Miyake Y, Shiraha H, Iwasaki Y, Shiratori Y, Yamamoto K (2010). Time-dependent analysis of predisposing factors for the recurrence of hepatocellular carcinoma. Liver Int.

[11] Nakazawa T, Hidaka H, Takada J, Okuwaki Y, Tanaka Y, Watanabe M, Shibuya A, Minamino T, Kokubu S, Koizumi W (2013). Early increase in alpha-fetoprotein for predicting unfavorable clinical outcomes in patients with advanced hepatocellular carcinoma treated with sorafenib. Eur J Gastroenterol Hepatol.

[12] Arai T, Kobayashi A, Ohya A, Takahashi M, Yokoyama T, Shimizu A, Motoyama H, Furusawa N, Notake T, Kitagawa N, Sakai H, Imamura H, Kadoya M, Miyagawa SI (2014). Assessment of treatment outcomes based on tumor marker trends in patients with recurrent hepatocellular carcinoma undergoing trans-catheter arterial chemo-embolization. Int J Clin Oncol.

[13] Hiraoka A, Ishimaru Y, Kawasaki H, Aibiki T, Okudaira T, Toshimori A, Kawamura T, Yamago H, Nakahara H, Suga Y, Azemoto N, Miyata H, Miyamoto Y, Ninomiya T, Hirooka M, Abe M, Matsuura B, Hiasa Y, Michitaka K (2015). Tumor markers AFP, AFP-L3, and DCP in hepatocellular carcinoma refractory to Transcatheter arterial chemoembolization. Oncology.

[14] Eisenhauer EA, Therasse P, Bogaerts J, Schwartz LH, Sargent D, Ford R, Dancey J, Arbuck S, Gwyther S, Mooney M, Rubinstein L, Shankar L, Dodd L, Kaplan R, Lacombe D, Verweij J (2009). New response evaluation criteria in solid tumours: revised RECIST guideline (version 1.1). Eur J Cancer.

[15] Johnson PJ, Berhane S, Kagebayashi C, Satomura S, Teng M, Reeves HL, O'Beirne J, Fox R, Skowronska A, Palmer D, Yeo W, Mo F, Lai P, Iñarrairaegui M, Chan SL, Sangro B, Miksad R, Tada T, Kumada T, Toyoda H (2015). Assessment of liver function in patients with hepatocellular carcinoma: a new evidence-based approach-the ALBI grade. J Clin Oncol.

[16] Cheng AL, Hsu C, Chan SL, Choo SP, Kudo M (2020). Challenges of combination therapy with immune checkpoint inhibitors for hepatocellular carcinoma. J Hepatol.

[17] Yau T, Kang YK, Kim TY, El-Khoueiry AB, Santoro A, Sangro B, Melero I, Kudo M, Hou MM, Matilla A (2020). Efficacy and safety of Nivolumab plus Ipilimumab in patients with advanced hepatocellular carcinoma previously treated with Sorafenib: the CheckMate 040 randomized clinical trial. JAMA Oncol.

[18] Zhu XD, Sun HC (2019). Emerging agents and regimens for hepatocellular carcinoma. J Hematol Oncol.

[19] Pinter M, Jain RK, Duda DG (2021). The current landscape of immune checkpoint blockade in hepatocellular carcinoma: a review. JAMA Oncol.

[20] Diem S, Kasenda B, Spain L, Martin-Liberal J, Marconcini R, Gore M, Larkin J (2016). Serum lactate dehydrogenase as an early marker for outcome in patients treated with anti-PD-1 therapy in metastatic melanoma. Br J Cancer.

[21] Nakamura Y, Kitano S, Takahashi A, Tsutsumida A, Namikawa K, Tanese K, Abe T, Funakoshi T, Yamamoto N, Amagai M, Yamazaki N (2016). Nivolumab for advanced melanoma: pretreatment prognostic factors and early outcome markers during therapy. Oncotarget.

[22] Tanizaki J, Haratani K, Hayashi H, Chiba Y, Nakamura Y, Yonesaka K, Kudo K, Kaneda H, Hasegawa Y, Tanaka K, Takeda M, Ito A, Nakagawa K (2018). Peripheral blood biomarkers associated with clinical outcome in non-small cell lung Cancer patients treated with Nivolumab. J Thorac Oncol.

[23] Dharmapuri S, Ozbek U, Lin JY, Sung M, Schwartz M, Branch AD, Ang C (2020). Predictive value of neutrophil to lymphocyte ratio and platelet to lymphocyte ratio in advanced hepatocellular carcinoma patients treated with anti-PD-1 therapy. Cancer Med.

[24] Feun LG, Li YY, Wu C, Wangpaichitr M, Jones PD, Richman SP, Madrazo B, Kwon D, Garcia-Buitrago M, Martin P, Hosein PJ, Savaraj N (2019). Phase 2 study of pembrolizumab and circulating biomarkers to predict anticancer response in advanced, unresectable hepatocellular carcinoma. Cancer.

[25] Inagaki Y, Tang W, Makuuchi M, Hasegawa K, Sugawara Y, Kokudo N (2011). Clinical and molecular insights into the hepatocellular carcinoma tumour marker des-gamma-carboxyprothrombin. Liver Int.

[26] Saeki I, Yamasaki T, Tanabe N, Iwamoto T, Matsumoto T, Urata Y, Hidaka I, Ishikawa T, Takami T, Yamamoto N, Uchida K, Terai S, Sakaida I (2015). A new therapeutic assessment score for advanced hepatocellular carcinoma patients receiving hepatic arterial infusion chemotherapy. PLoS One.

[27] Lim TS, Rhee H, Kim GM, Kim SU, Kim BK, Park JY, Ahn SH, Han KH, Choi JY, Kim DY (2019). Alpha-fetoprotein, des-gamma-Carboxy prothrombin, and modified RECIST response as predictors of survival after Transarterial Radioembolization for hepatocellular carcinoma. J Vasc Interv Radiol.

[28] Kodama K, Kawaoka T, Namba M, Uchikawa S, Ohya K, Morio K, Nakahara T, Murakami E, Yamauchi M, Hiramatsu A, Imamura M, Chayama K, Aikata H (2019). Correlation between early tumor marker response and imaging response in patients with advanced hepatocellular carcinoma treated with Lenvatinib. Oncology.

[29] Dal Bello MG, Filiberti RA, Alama A, Orengo AM, Mussap M, Coco S, Vanni I, Boccardo S, Rijavec E, Genova C, Biello F, Barletta G, Rossi G, Tagliamento M, Maggioni C, Grossi F (2019). The role of CEA, CYFRA21-1 and NSE in monitoring tumor response to Nivolumab in advanced non-small cell lung cancer (NSCLC) patients. J Transl Med.

[30] Henderson NC, Arnold TD, Katamura Y, Giacomini MM, Rodriguez JD, McCarty JH, Pellicoro A, Raschperger E, Betsholtz C, Ruminski PG (2013). Targeting of alphav integrin identifies a core molecular pathway that regulates fibrosis in several organs. Nat Med.

[31] Hernandez-Gea V, Friedman SL (2011). Pathogenesis of liver fibrosis. Annu Rev Pathol.

[32] Affo S, Yu LX, Schwabe RF (2017). The role of Cancer-associated fibroblasts and fibrosis in liver Cancer. Annu Rev Pathol.

[33] Wong JSL, Kwok GGW, Tang V, et al. Ipilimumab and nivolumab/pembrolizumab in advanced hepatocellular carcinoma refractory to prior immune checkpoint inhibitors. J Immunother Cancer. 2021;9(2). 10.1136/jitc-2020-001945. 10.1136/jitc-2020-001945 PMC7875295 33563773

[34] Kim BK, Ahn SH, Seong JS, Park JY, Kim DY, Kim JK, Lee DY, Lee KH, Han KH (2011). Early alpha-fetoprotein response as a predictor for clinical outcome after localized concurrent chemoradiotherapy for advanced hepatocellular carcinoma. Liver Int.

[35] Mei J, Li SH, Li QJ, Sun XQ, Lu LH, Lin WP, Zheng L, Chen MS, Shi M, Wei W, Guo RP (2021). Anti-PD-1 immunotherapy improves the efficacy of hepatic artery infusion chemotherapy in advanced hepatocellular carcinoma. J Hepatocell Carcinoma.

[36] He MK, Liang RB, Zhao Y, Xu YJ, Chen HW, Zhou YM, Lai ZC, Xu L, Wei W, Zhang YJ (2021). Lenvatinib, toripalimab, plus hepatic arterial infusion chemotherapy versus lenvatinib alone for advanced hepatocellular carcinoma. Ther Adv Med Oncol.

